# Prevalence of *Escherichia albertii* in Raccoons (*Procyon lotor*), Japan

**DOI:** 10.3201/eid2606.191436

**Published:** 2020-06

**Authors:** Atsushi Hinenoya, Keigo Nagano, Sharda P. Awasthi, Noritoshi Hatanaka, Shinji Yamasaki

**Affiliations:** Osaka Prefecture University, Osaka, Japan

**Keywords:** *Escherichia albertii*, reservoir, raccoon, bacteria, Japan, zoonoses, enteric infections

## Abstract

Natural reservoirs of *Escherichia albertii* remain unclear. In this study, we detected *E. albertii* by PCR in 248 (57.7%) of 430 raccoons from Osaka, Japan, and isolated 143 *E. albertii* strains from the 62 PCR-positive samples. These data indicate that raccoons could be a natural reservoir of *E. albertii* in Japan.

*Escherichia albertii* is a gram-negative facultative anaerobic bacterium and an emerging human enteropathogen. This bacterium belongs to the group of attaching and effacing pathogens, which can form pedestal-structured lesions on intestinal epithelium by using an *eae*-encoded adhesin called intimin and a type 3 secretion system. *E. albertii* commonly carries cytolethal distending toxin genes; in addition, certain strains carry Shiga toxin 2 (*stx2a*, *stx2f*) genes (*1*), suggesting that *E. albertii* has a potential to cause severe diseases such as hemorrhagic colitis and hemolytic uremic syndrome in humans, similar to Shiga toxin–producing *E. coli*. An increase in human outbreaks and sporadic cases of *E. albertii* have been reported recently from several countries, including Japan (*1**–**3*). However, the reservoir and transmission routes of *E. albertii* to humans have not yet been identified. We surveyed wild raccoons (*Procyon lotor*) captured in Osaka, Japan, for the presence of *E. albertii* to determine if raccoons could be a reservoir of *E. albertii* in Japan.

## The Study

We collected 430 rectal swabs from wild raccoons in Osaka during 2016–2017 (Appendix). To determine the presence of *E. albertii*, we first subjected fecal specimens to an *E. albertii*–specific *cdt* (*Eacdt*) gene-based PCR assay (*4*) after enrichment in tryptic soy broth. Of these 430 specimens, 248 (57.7%) yielded a 449-bp PCR amplicon specific for *E. albertii* (Table 1). By using XRM-MacConkey agar developed for the isolation of *E. albertii* (Appendix), we isolated and selected 143 *E. albertii* isolates from the 62 PCR-positive specimens (1–8 isolates/sample) with species identity confirmed by 2 different *E. albertii*–specific PCRs using primers targeting *Eacdt* (*4*), and *yejH* and *yejK* (*5*).

**Table 1:** Prevalence of *Escherichia albertii* in Japanese wild raccoon fecal specimens and number of isolates

Sampling year and month	No. specimens	No. (%) PCR positive	No. specimens from which *E. albertii* was isolated	No. *E. albertii* isolates
2016				
Jun	57	25 (43.9)	7	17
Jul	55	34 (61.8)	4	8
Aug	22	14 (63.6)	0	0
Sep	7	2 (28.6)	0	0
Oct	8	3 (37.5)	0	0
Dec	14	7 (50.0)	0	0
2017				
Feb	3	2 (66.7)	0	0
Mar	16	3 (18.8)	0	0
Jul	88	56 (63.6)	14	21
Aug	104	63 (60.6)	21	56
Sep	56	39 (69.6)	16	41
Total	430	248 (57.7)	62	143

To determine the phylogenetic relationships among the isolates, we performed pulsed-field gel electrophoresis (PFGE) using *Xba*I-digested genomic DNA. The 143 isolates showed 59 pulsotypes (Figure), indicating that *E. albertii* isolates from raccoons were genetically diverse. We obtained 2–7 *E. albertii* isolates, which were determined to be clonal by PFGE, from 26 of 29 raccoons. The isolates from each of 3 raccoons (R305, R318, R419) showed 2–3 different DNA fingerprints with >3 bands different from each other, indicating that multiclonal *E. albertii* strains coexisted in the intestine of each of these 3 raccoons (Figure). In addition, we frequently observed that the isolates from different raccoons displayed exactly the same PFGE pattern (e.g., R7, R8, and R335; Figure), although the raccoons were usually captured in different locations in Osaka.

**Figure:**
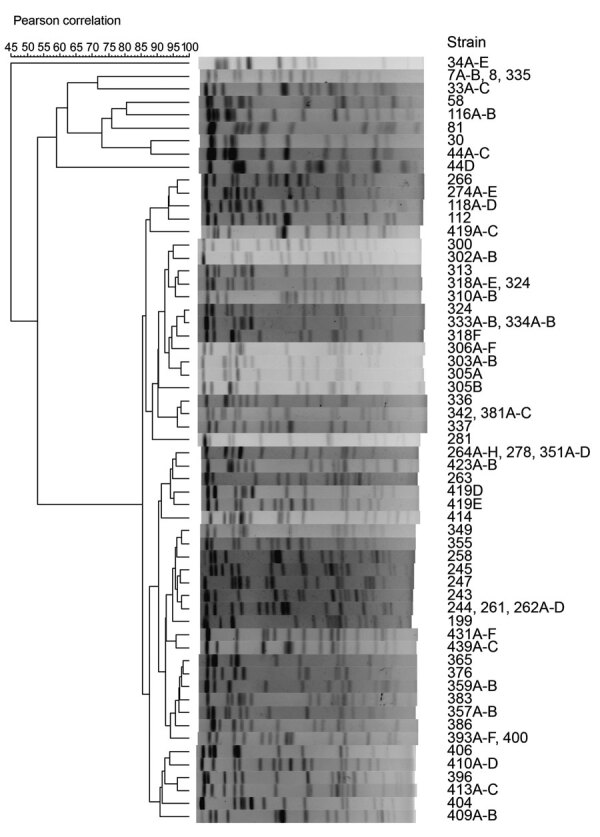
Phylogenetic analysis of raccoon *Escherichia albertii* strains by pulsed-field gel electrophoresis (PFGE). *Xba*I-digested genomic DNA of 143 raccoon *E. albertii* strains isolated in this study were analyzed by PFGE. The dendrogram was constructed based on DNA fingerprints obtained (Appendix). The number in each strain name represents a specific raccoon identification number.

To evaluate the human pathogenic potential of *E. albertii* isolated from raccoons, we selected 1 isolate from each pulsotype (n = 59) and tested for the presence of virulence determinants in clinical *E. albertii* isolates (Appendix). We detected the *eae* gene in 59 strains (100%), *Eccdt-*I in 5 strains (8.5%), and *stx2f* genes in 2 strains (3.4%). By sequencing the entire *eae* gene in 59 strains (Appendix), we determined the intimin subtypes to be ρ (n = 8), ι2 (n = 5), ο (n = 4), ς (n = 4), γ5 (n = 2), ξ (n = 2), α8 (n = 1), β3 (n = 1), and unknown (n = 32) (Appendix Table 3). Among the 32 unknown subtypes, 16 were grouped into the 5 subtypes (N1–N5) that were recently identified in clinical *E. albertii* strains from Japan (*6*). Two subtypes were homologous to those identified in clinical *E. albertii* strains 1251–6/89, 2 were homologous to strain 4281–7/89 (*7*), and 3 were homologous to those identified in strain 2013C-4143 (GenBank accession no. CP030787). We also identified 2 novel subtypes (UT1 and UT2; Appendix Table 3) in the remaining 9 strains; each showed <95% nt and aa identities with any known subtypes. We identified complete *stx2f* genes in the *stx2f* gene-positive strains RAC-199 and RAC-247. The culture supernatants caused Vero cell deaths, which were neutralized by anti-Stx2fA serum, indicating that both strains produced biologically active Stx2f. The toxin activity was enhanced in the presence of mitomycin C, indicating that the *stx2f* genes could be located on inducible prophage genomes (Table 2). The fold change of Stx2f production by mitomycin C in the strains RAC-199 and RAC-247 were comparable to that of Stx2 production in human clinical strains *E. albertii* AKT5 and EHEC O157:H7 Sakai. These data suggest that the *E. albertii* strains isolated from raccoons have a potential to cause serious human diseases.

**Table 2:** Stx2 production by *stx2f* gene-positive *Escherichia albertii* raccoon strains.

Species	Strains	Toxin gene	Mitomycin C	Toxin titer*	Neutralization†
E. albertii	*RAC199*	stx2f	*Negative*	*4*	*Yes*
			*Positive*	*512*	*Yes*
	*RAC247*	stx2f	*Negative*	*32*	*Yes*
			*Positive*	*1,024*	*Yes*
	*AKT5*	stx2f	*Negative*	*512*	*Yes*
			*Positive*	*32,768*	*Yes*
E. coli	*Sakai*	stx1, stx2a	*Negative*	*256*	*Not done*
			*Positive*	*16,384*	*Not done*
	*C600*	*None*	*Negative*	*<1*	*Not done*
			*Positive*	*<1*	*Not done*
*Reciprocal of highest dilution that resulted in death in >50% of cells is shown as toxin titer. †Neutralization of cytotoxic effect by anti-Stx2f rabbit serum. Filtrated culture supernatants in LB-broth with and without mitomycin C were used as toxin samples.					

## Conclusions

*E. albertii* is known to be an emerging zoonotic pathogen and has been isolated from various animals, such as pigs, cats, and birds (*6*,*8*,*9*). Although much effort has been devoted to identify the natural reservoir, *E. albertii* was not detected in vertebrate animals such as fish (n = 138), amphibians (n = 106), reptiles (n = 447), and mammals (n = 1,063) (*3*) but was found in 1.4% (9/634) of birds in Australia and 0.9% (9/1,204) of birds in Korea. Thus, the natural reservoir of *E. albertii* is still unclear; this information would be essential to determine transmission dynamics and prevent *E. albertii* infections. Given that patients in clinical outbreaks in Japan might be infected thorough waters (spring and well waters) or vegetables, but not meats (*3*), the natural reservoir of *E. albertii* might not be major food animals (e.g., cattle and chickens, the reservoirs for Shiga toxin–producing *E. coli* and *Campylobacter jejuni*, respectively). Another possibility is wild animals, which may contaminate environmental water and vegetables. Among the wild animals, the raccoon is a synanthropic animal with the ability to reside in a wide range of habitats, including agricultural, forested, and urban areas. Raccoons are omnivorous and forage within vegetable fields. They also prefer riparian environments. Furthermore, raccoons are known to carry various pathogenic microorganisms (*10**–**12*). Thus, they can contaminate vegetables and waters with pathogens, possibly including *E. albertii*, leading to human infections. Therefore, we performed a survey targeting raccoons and found that *E. albertii* was highly prevalent (248/430; 57.7%) in wild raccoons in Japan, indicating that carriage of *E. albertii* by raccoons is not incidental. The *E. albertii* strains isolated from raccoons also possessed virulence determinants (*eae*, *Eacdt, Eccdt-I*, or *stx2f*) present in human clinical strains. Almost all the intimin subtypes of the raccoon strains were those identified in human clinical *E. albertii* strains. Two strains produced functional Stx2f, which may have a potential to cause severe diseases in humans. Taken together, these data suggested that raccoons constitute a major reservoir of *E. albertii* and could be a source of human infection in Japan.

Raccoons originated from North America and were introduced as pets or game animals into other countries, including Japan and countries in Europe. Some of these have escaped and settled in the wild. The number of raccoons has increased because of their adaptability to various environments, omnivorous feeding habits, high reproductive potential, and lack of predators in the environment (*13*). 

In addition to Japan, *E. albertii* has been clinically isolated in other countries where raccoons reside (*9*,*14*,*15*). Interactions between raccoons and other animals, such as wild mice and wild boars, can also be possible. Therefore, further epidemiologic studies to survey raccoons and other wild animals in Osaka, other areas of Japan, and other countries are highly warranted to evaluate the significance of raccoons as a natural reservoir of *E. albertii*.

Appendix Additional information about the study of *Escherichia albertii* in raccoons, Japan.
