# Supplementary material for: Prevalence of Escherichia albertii in Raccoons (Procyon lotor), Japan
Source: Emerg Infect Dis. 2020 Jun;26(6):1304–7. doi: 10.3201/eid2606.191436 (PMC7258444; doi:10.3201/eid2606.191436)
Supplement: Appendix — Additional information about the study of Escherichia albertii in raccoons, Japan. [file 19-1436-Techapp-s1.pdf]

Article DOI: https://doi.org/10.3201/eid2606.191436

Prevalence of *Escherichia albertii* in Raccoons (*Procyon lotor*), Japan

Appendix

Sample Collection

We collected rectal specimens using cotton swabs (SEEDSWAB γ1, Eiken Chemical Co., http://www.eiken.co.jp) from 430 wild raccoons (*Procyon lotor*) in Osaka, Japan, during June 2016–March 2017 (n = 182) and July–September 2017 (n = 248). All the raccoons seemed to be asymptomatic. Raccoons were captured to exterminate them throughout the year in Osaka. The samples were transported to the laboratory at ambient temperature and processed within 6 hours of collection. Fecal sampling in the present study was approved by Osaka Prefectural Government and performed according to the Guidelines for Animal Experimentation of Osaka Prefectural Animal Protection and Livestock Division.

Detection of *Eacdt* Genes by PCR

We suspended rectal swabs in 1 mL of sterilized Dulbecco's phosphate-buffered saline (PBS). An aliquot (300 μL) of the suspension was inoculated into 3 mL of tryptic soy broth (Becton Dickinson, www.bd.com), and enriched them at 37°C for 14–16 h with shaking. We centrifuged 100 μL of the culture at 10,000 *g* at 4°C for 3 min. We suspended the resulting pellet in 85 μL of 50 mM NaOH, boiled it at 100°C for 10 min, and neutralized it by adding 15 μL of 1 M Tris-HCl buffer (pH 7.0). After centrifugation at 10,000 *g* at 4°C for 10 min, we subjected the supernatant to PCR analysis using a pair of *E. albertii* specific primers targeting *Eacdt* genes (Appendix Table 1).

Isolation and identification of *E. albertii*

We serially diluted the swab suspensions from PCR-positive specimens in PBS and spread 100 μL of each dilution on XRM-MacConkey agar, an *E. albertii*-selective medium (*1*), with composition of MacConkey agar base (Becton Dickinson) supplemented with 1% (w/v) each of xylose, rhamnose, and melibiose, and incubated them at 37°C for 20–24 hours. We examined colorless colonies (maximum 8 colonies) on the medium, which are typical feature of *E. albertii*, by PCR, targeting *Eacdt* genes. *Eacdt* gene-positive colonies were determined to be *E. albertii* by another *E. albertii*-specific PCR assay using a primer pair targeting *yejH* and *yejK* in *E. albertii*, which was developed by Ooka et al. (*2*).

Detection of Virulence Genes

We analyzed the presence of virulence genes by colony hybridization assay using ^{32}P-labeled DNA probes targeting *eae*, *stx1*, *stx2a*, *stx2f*, *Eccdt-IB*, and *Eccdt-IVB* under high stringent conditions, as described previously (*3*). When *stx2* and *cdt* genes were detected by the colony hybridization assay, subtype-specific PCRs for *stx2* (*4*), *Eccdt-I* and *Eccdt-IV* were carried out to determine their subtypes (Appendix Table 1). The entire nucleotide sequence of *stx2f* genes was determined as described previously (*5*). PCR amplification was done by Veriti Thermal cycler (Thermo Fisher Scientific, https://www.thermofisher.com) using TaKaRa Taq DNA polymerase (Takara Bio, https://www.takarabio.com). We sequenced the PCR products by cycle sequencing method using BigDye Terminator v1.1 and ABI 3130 Genetic Analyzer (Thermo Fisher Scientific).

To determine each intimin subtype, we determined the entire *eae* nucleotide sequence, as previously described (*5*). Predicted amino acid sequences of *eae* genes were aligned with those of the reference intimin subtypes by the Clustal W program of MEGA6 (https://www.megasoftware.net). The reference intimin subtypes used were from Hinenoya et al. (*5*). If intimin subtypes of *E. albertii* raccoon strains were determined to be untypable, the putative amino acid sequences were subjected to BLAST homology search using the tblastn module (http://blast.ncbi.nlm.nih.gov/Blast.cgi).

Pulsed-Field Gel Electrophoresis (PFGE)

We performed PFGE as described previously (*6*). Briefly, fresh bacterial cells were embedded in agarose plug and in situ lysis was carried out to isolate total genomic DNA. The genomic DNA embedded plug was subjected to restriction enzyme digestion with 30 U of *Xba*I (Takara Bio), and electrophoretic separation of the DNA fragments was done in 1% pulsed-field certified agarose (Bio-Rad Laboratories, https://www.bio-rad.com) on a CHEF Mapper PFGE (Bio-Rad) using 0.5x TBE buffer (45 mM Tris, 45 mM boric acid, 1 mM EDTA [pH 8.0]). Run conditions were generated by the autoalgorithm mode of the CHEF Mapper PFGE system for the sizes ranging between 20 and 300 kb, and the running time was 26.93 hours. *Xba*I-digested genomic DNA of *Salmonella* Braenderup strain H98121 was used as a molecular size marker. DNA fingerprints of *E. albertii* strains were interpreted based on Tenover's criteria (*7*) and analyzed using Fingerprinting II Software (Bio-Rad) to know their phylogenetic relationships.

Detection of Stx2f Production in *stx2f* Gene-Positive *E. albertii*

Production of Stx2f by *stx2f* gene-positive *E. albertii* strains was determined by Vero cells cytotoxicity assay, as previously described (*3*). We prepared crude toxin samples as follows: *E. albertii* was cultured in 3 mL of lysogenic broth (LB broth, Becton Dickinson) at 37°C for 14 hours. An aliquot of the culture was inoculated into 3 mL of fresh lysogenic broth

and cultured until early log phase (≈0.2 optical density at 600 nm). Mitomycin C (Kyowa Hakko Kirin, https://www.kyowakirin.com) was added to the culture at the final concentration of 0.5 μg/mL and further incubated at 37°C for 4 hours aerobically. Culture supernatant was passed through a sterile filter with 0.22-μm pore size (Merck Millipore, https://www.emdmillipore.com), and the filtrate was subjected to cytotoxicity assay. Neutralization assay of the toxin activity was also carried out using anti-Stx2fA rabbit serum (*8*), which was preincubated with crude toxin samples at 37°C for 30 min. The mixture was applied to the cytotoxicity assay.

Nucleotide Sequence Accession Numbers

All nucleotide sequences obtained in this study were registered into the DNA Data Bank of Japan database. The accession numbers are LC504574–LC504632 (for *eae* genes) and LC504633–LC504634 (for *stx2f* genes).

References

1. Hinenoya A, Nagano K, Okuno K, Nagita A, Hatanaka N, Awasthi SP, et al. Development of XRM-MacConkey agar selective medium for the isolation of *Escherichia albertii.* Diagn Microbiol Infect Dis. 2020;115006. https://doi.org/10.1016/j.diagmicrobio.2020.115006

2. Ooka T, Ogura Y, Katsura K, Seto K, Kobayashi H, Kawano K, et al. Defining the genome features of *Escherichia albertii*, an emerging enteropathogen closely related to *Escherichia coli.* Genome Biol Evol. 2015;7:3170–9. PubMed

3. Hinenoya A, Ichimura H, Awasthi SP, Yasuda N, Yatsuyanagi J, Yamasaki S. Phenotypic and molecular characterization of *Escherichia albertii*: Further surrogates to avoid potential laboratory misidentification. Int J Med Microbiol. 2019;309:108–15. PubMed https://doi.org/10.1016/j.ijmm.2018.12.003

4. Beutin L, Miko A, Krause G, Pries K, Haby S, Steege K, et al. Identification of human-pathogenic strains of Shiga toxin-producing *Escherichia coli* from food by a combination of serotyping and molecular typing of Shiga toxin genes. Appl Environ Microbiol. 2007;73:4769–75. PubMed https://doi.org/10.1128/AEM.00873-07

5. Hinenoya A, Yasuda N, Mukaizawa N, Sheikh S, Niwa Y, Awasthi SP, et al. Association of cytolethal distending toxin-II gene-positive *Escherichia coli* with *Escherichia albertii*, an emerging enteropathogen. Int J Med Microbiol. 2017;307:564–71. PubMed https://doi.org/10.1016/j.ijmm.2017.08.008

6. Shima K, Wu Y, Sugimoto N, Asakura M, Nishimura K, Yamasaki S. Comparison of a PCR-restriction fragment length polymorphism (PCR-RFLP) assay to pulsed-field gel electrophoresis to determine the effect of repeated subculture and prolonged storage on RFLP patterns of Shiga toxin-producing *Escherichia coli* O157:H7. J Clin Microbiol. 2006;44:3963–8. PubMed https://doi.org/10.1128/JCM.00717-06

7. Tenover FC, Arbeit RD, Goering RV, Mickelsen PA, Murray BE, Persing DH, et al. Interpreting chromosomal DNA restriction patterns produced by pulsed-field gel electrophoresis: criteria for bacterial strain typing. J Clin Microbiol. 1995;33:2233–9. PubMed https://doi.org/10.1128/JCM.33.9.2233-2239.1995

8. Hinenoya A, Yasuda N, Hibino T, Shima A, Nagita A, Tsukamoto T, et al. Isolation and characterization of an *Escherichia albertii* strain producing three different toxins from a child with diarrhea. Jpn J Infect Dis. 2017;70:252–7. PubMed https://doi.org/10.7883/yoken.JJID.2016.186

9. Hinenoya A, Ichimura H, Yasuda N, Harada S, Suzuki M, Iijima Y, et al. Development of a cytolethal distending toxin (*cdt*) gene-based PCR for the detection and identification of *Escherichia albertii.* Diagn Microbiol Infect Dis. 2019;95:119–24. PubMed https://doi.org/10.1016/j.diagmicrobio.2019.04.018

10. Hinenoya A, Naigita A, Ninomiya K, Asakura M, Shima K, Seto K, et al. Prevalence and characteristics of cytolethal distending toxin-producing *Escherichia coli* from children with diarrhea in Japan. Microbiol Immunol. 2009;53:206–15. PubMed https://doi.org/10.1111/j.1348-0421.2009.00116.x

Appendix Table 1. PCR primers for detection of *E. albertii*, and differentiating *Eccdt-I* and *Eccdt-IV* genes;.PCR conditions (30 cycles).

Target gene	Primer name	Sequence (5'-3')	PCR conditions (30 cycles)			Amplicon size (bp)	Reference
			Denaturing	Annealing	Extension		
Eacdt	EaCDTsp-F2	GCTTAACTGGATGATTCTTG	94°C, 30 sec	50°C, 30 sec	72°C, 60 sec	449	(*9*)
	EaCDTsp-R2	CTATTTCCCATCCAATAGTCT					
Eccdt-IA	EcCDT1A-F	GAT CGG TGA TTC ACC TTC	94°C, 30 sec	50°C, 30 sec	72°C, 60 sec	499	This study
	EcCDT1A-R	TTT CTC AAG GGT GAT TGT AA					
Eccdt-IB	EcCDT1B-F	GAT TTT GCC GGG TAT TTC T	94°C, 30 sec	50°C, 30 sec	72°C, 60 sec	640	This study
	EcCDT1B-R	TCA AGA ACA CCA CCA CTG					
Eccdt-IC	EcCDT1C-F	TAC TGC TGA CAG GTT GTG	94°C, 30 sec	50°C, 30 sec	72°C, 60 sec	540	This study
	EcCDT1C-R	CAG CTC GTT AAT GGA GAC					(*10*)
Eccdt-IVA	EcCDT4A-F	TCT CCA ACA TTT GGG AG	94°C, 30 sec	50°C, 30 sec	72°C, 60 sec	427	(*10*)
	EcCDT4A-R	CTT TTG CAC CAG GAC AC					This study
Eccdt-IVB	EcCDT4B-F	ACC ATC TTC AGC TAC ACT A	94°C, 30 sec	50°C, 30 sec	72°C, 60 sec	286	This study
	EcCDT4B-R	GCT CCA GAA TCT ATA CCT					
Eccdt-IVC	EcCDT4C-F	TCA GAA ACC CTG TAG GTC	94°C, 30 sec	50°C, 30 sec	72°C, 60 sec	202	This study
	EcCDT4C-R	GTA AAT AAT GCA TTG CGA TTG					

Appendix Table 2. Information about raccoons from which *Escherichia albertii* strains were isolated.

Strain	Raccoon ID	City captured*	Month of sampling
RAC-7A	R7	A	2016 Jun
RAC-7B			
RAC-8	R8	B	2016 Jun
RAC-30	R30	C	2016 Jun
RAC-33A	R33	B	2016 Jun
RAC-33B			
RAC-33C			
RAC-34A	R34	B	2016 Jun
RAC-34B			
RAC-34C			
RAC-34D			
RAC-34E			
RAC-44A	R44	B	2016 Jun
RAC-44B			
RAC-44C			
RAC-44D			
RAC-58	R58	D	2016 Jun
RAC-81	R81	A	2016 Jul
RAC-112	R112	E	2016 Jul
RAC-116A	R116	A	2016 Jul
RAC-116B			
RAC-118A	R118	B	2016 Jul
RAC-118B			
RAC-118C			
RAC-118D			
RAC-199	R199	F	2017 Jul
RAC-243	R243	G	2017 Jul
RAC-244	R244	C	2017 Jul
RAC-245	R245	C	2017 Jul
RAC-247	R247	H	2017 Jul
RAC-258	R258	A	2017 Jul
RAC-261	R261	I	2017 Jul
RAC-262A	R262	J	2017 Jul
RAC-262B			
RAC-262C			
RAC-262D			
RAC-263	R263	D	2017 Jul
RAC-264A	R264	H	2017 Jul
RAC-264B			
RAC-264C			
RAC-264D			
RAC-264E			
RAC-264F			
RAC-264G			
RAC-264H			
RAC-266	R266	C	2017 Jul
RAC-274A	R274	F	2017 Jul
RAC-274B			
RAC-274C			
RAC-274D			
RAC-274E			
RAC-278A	R278	G	2017 Jul
RAC-278B			
RAC-278C			
RAC-278D			
RAC-278E			
RAC-278F			
RAC-278G			
RAC-281	R281	C	2017 Jul
RAC-300	R300	K	2017 Aug
RAC-302A	R302	L	2017 Aug
RAC-302B			
RAC-303A	R303	E	2017 Aug
RAC-303B			
RAC-305A	R305	K	2017 Aug
RAC-305B			
RAC-306A	R306	K	2017 Aug
RAC-306B			
RAC-306C			
RAC-306D			
RAC-306E			
RAC-306F			
RAC-310A	R310	G	2017 Aug
RAC-310B			
RAC-313	R313	G	2017 Aug

Strain	Raccoon ID	City captured*	Month of sampling
RAC-318A	R318	M	2017 Aug
RAC-318B			
RAC-318C			
RAC-318D			
RAC-318E			
RAC-318F			
RAC-324	R324	C	2017 Aug
RAC-333A	R333	J	2017 Aug
RAC-333B			
RAC-334A	R334	F	2017 Aug
RAC-334B			
RAC-335	R335	F	2017 Aug
RAC-336	R336	D	2017 Aug
RAC-337	R337	D	2017 Aug
RAC-342	R342	G	2017 Aug
RAC-349	R349	N	2017 Aug
RAC-351A	R351	A	2017 Aug
RAC-351B			
RAC-351C			
RAC-351D			
RAC-355	R355	A	2017 Aug
RAC-357A	R357	M	2017 Aug
RAC-357B			
RAC-359A	R359	C	2017 Aug
RAC-359B			
RAC-365	R365	H	2017 Aug
RAC-376	R376	D	2017 Aug
RAC-381A	R381	K	2017 Sep
RAC-381B			
RAC-381C			
RAC-382	R382	O	2017 Sep
RAC-386	R386	P	2017 Sep
RAC-393A	R393	B	2017 Sep
RAC-393B			
RAC-393C			
RAC-393D			
RAC-393E			
RAC-393F			
RAC-396	R396	N	2017 Sep
RAC-400	R400	E	2017 Sep
RAC-404	R404	L	2017 Sep
RAC-406	R406	K	2017 Sep
RAC-409A	R409	A	2017 Sep
RAC-409B			
RAC-410A	R410	N	2017 Sep
RAC-410B			
RAC-410C			
RAC-410D			
RAC-413A	R413	C	2017 Sep
RAC-413B			
RAC-413C			
RAC-414	R414	I	2017 Sep
RAC-419A	R419	A	2017 Sep
RAC-419B			
RAC-419C			
RAC-419D			
RAC-419E			
RAC-423A	R423	B	2017 Sep
RAC-423B			
RAC-431A	R431	H	2017 Sep
RAC-431B			
RAC-431C			
RAC-431D			
RAC-431E			
RAC-431F			
RAC-439A	R439	H	2017 Sep
RAC-439B			
RAC-439C			

*City names are coded by alphabetical letters (A–P).

Appendix Table 3. Detailed information and characteristics of *E. albertii* raccoon strains.

	Virulence genes				
Strain*	*Eacdt*	*eae*	*Eccdt-I*	*stx2f*	Intimin subtypes
RAC-7A	+	+	–	–	rho
RAC-30A	+	+	–	–	xi
RAC-33A	+	+	–	–	omicron
RAC-34A	+	+	–	–	beta3
RAC-44A	+	+	–	–	xi

Strain*	Virulence genes				Intimin subtypes
	Eacdt	*eae*	*Eccdt-I*	*stx2f*	
RAC-44D	+	+	+	–	gamma5
RAC-58	+	+	–	–	N4
RAC-81A	+	+	–	–	sigma
RAC-112	+	+	–	–	N5
RAC-116A	+	+	–	–	N1.3
RAC-118A	+	+	–	–	alpha8
RAC-199	+	+	–	+	N1.2
RAC-243	+	+	–	–	N2
RAC-244	+	+	–	–	N5
RAC-245	+	+	–	–	Unknown (2013C-4143)
RAC-247	+	+	+	+	gamma5
RAC-258	+	+	–	–	iota2
RAC-263	+	+	–	–	N2
RAC-264A	+	+	–	–	Unknown (1261-6/89)
RAC-266	+	+	–	–	rho
RAC-274A	+	+	+	–	N3
RAC-281	+	+	–	–	sigma
RAC-300	+	+	–	–	rho
RAC-302A	+	+	–	–	sigma
RAC-303A	+	+	+	–	N3
RAC-305A	+	+	+	–	unknown (1261-6/89)
RAC-305B	+	+	–	–	N3
RAC-306A	+	+	–	–	Unknown (4281-7/89)
RAC-310A	+	+	–	–	omicron
RAC-313	+	+	–	–	UT1
RAC-318A	+	+	–	–	UT2
RAC-318F	+	+	–	–	UT2
RAC-324	+	+	–	–	N3
RAC-333A	+	+	–	–	UT1
RAC-336	+	+	–	–	N3
RAC-337	+	+	–	–	N5
RAC-342	+	+	–	–	sigma
RAC-349	+	+	–	–	Unknown (4281-7/89)
RAC-355	+	+	–	–	rho
RAC-357A	+	+	–	–	UT1
RAC-359A	+	+	–	–	UT1
RAC-365	+	+	–	–	rho
RAC-376	+	+	–	–	iota2
RAC-383	+	+	–	–	N4
RAC-386	+	+	–	–	UT2
RAC-393A	+	+	–	–	N5
RAC-396	+	+	–	–	Unknown (2013C-4143)
RAC-404	+	+	–	–	UT1
RAC-406	+	+	–	–	rho
RAC-409A	+	+	–	–	iota2
RAC-410A	+	+	–	–	rho
RAC-413A	+	+	–	–	Unknown (2013C-4143)
RAC-414	+	+	–	–	iota2
RAC-419A	+	+	–	–	iota2
RAC-419D	+	+	–	–	UT1
RAC-419E	+	+	–	–	rho
RAC-423A	+	+	–	–	N4
RAC-431A	+	+	–	–	omicron
RAC-439A	+	+	–	–	omicron

*RAC, number, letter (A–F) indicate raccoon, raccoon ID, and *E. albertii* colony ID, respectively. Strains with identical numbers were isolated from the same raccoons. +, present; –, not present.
